# Progenitor Renin Lineage Cells are not involved in the regeneration of glomerular endothelial cells during experimental renal thrombotic microangiopathy

**DOI:** 10.1371/journal.pone.0196752

**Published:** 2018-05-17

**Authors:** Leo Ruhnke, Jan Sradnick, Moath Al-Mekhlafi, Michael Gerlach, Florian Gembardt, Bernd Hohenstein, Vladimir T. Todorov, Christian Hugo

**Affiliations:** Division of Nephrology, Department of Internal Medicine III, University Hospital Carl Gustav Carus, TU Dresden, Fetscherstrasse 74, Dresden, Germany; Anatomy, SWITZERLAND

## Abstract

Endothelial cells (EC) frequently undergo primary or secondary injury during kidney disease such as thrombotic microangiopathy or glomerulonephritis. Renin Lineage Cells (RLCs) serve as a progenitor cell niche after glomerular damage in the adult kidney. However, it is not clear whether RLCs also contribute to endothelial replenishment in the glomerulus following endothelial injury. Therefore, we investigated the role of RLCs as a potential progenitor niche for glomerular endothelial regeneration. We used an inducible tet-on triple-transgenic reporter strain mRen-rtTAm2/LC1/LacZ to pulse-label the renin-producing RLCs in adult mice. Unilateral kidney EC damage (EC model) was induced by renal artery perfusion with concanavalin/anti-concanavalin. In this model glomerular EC injury and depletion developed within 1 day while regeneration occurred after 7 days. LacZ-labelled RLCs were restricted to the juxtaglomerular compartment of the afferent arterioles at baseline conditions. In contrast, during the regenerative phase of the EC model (day 7) a subset of LacZ-tagged RLCs migrated to the glomerular tuft. Intraglomerular RLCs did not express renin anymore and did not stain for glomerular endothelial or podocyte cell markers, but for the mesangial cell markers α8-integrin and PDGFRβ. Accordingly, we found pronounced mesangial cell damage parallel to the endothelial injury induced by the EC model. These results demonstrated that in our EC model RLCs are not involved in endothelial regeneration. Rather, recruitment of RLCs seems to be specific for the repair of the concomitantly damaged mesangium.

## Introduction

Endothelial cell (EC) injury is a pivotal pathophysiological factor involved in many kidney diseases, such as thrombotic microangiopathy, lupus nephritis, membrano-proliferative and C3 dominant glomerulonephritis as well as diabetic nephropathy. In addition, progressive capillary rarefaction associated with chronic renal disease progression in numerous experimental and human kidney diseases [1]. Therefore, preservation and restoration of EC function represent central therapeutic approaches to prevent/inhibit chronic renal disease progression [2, 3]. Although the EC compartment shows remarkable regenerative capacity [4], the exact mechanisms of endothelial recovery after injury remain largely unknown. Performing combined kidney and bone marrow transplantation experiments we previously demonstrated that extrarenal cells are not involved in the repair of damaged renal endothelium in a concanavalin-A/anti-concanavalin-A model of renal thrombotic microangiopathy (EC model) and ischemia/reperfusion acute kidney injury [5]. While other experimental renal disease models focusing on the podocyte, glomerular basement membrane (GBM) or mesangium [6–8] may be accompanied by some less defined EC injury, our concanavalin-based model is the only setup, in which a severe loss and subsequent recovery of the renal endothelium has been demonstrated [1, 5, 9, 10].

Therefore, local mechanisms must be primarily responsible for endothelial regeneration in the adult kidney [11]. Both EC proliferation and recruitment of intrarenal progenitors are possible pathways in renal endothelial regeneration. In our earlier study we reported that intrarenal proliferation of surviving EC after renal injury may only partially account for the successful regeneration [5]. Therefore, intrarenal progenitor niches are likely to be involved in EC replenishment in this model. A growing body of studies by us and others demonstrated that Renin Lineage Cells (RLCs) serve as a local precursor cell pool in the kidney [6–8, 12]. Furthermore, we recently reported that RLCs are constantly filled up by non-RLCs through regulated *de novo* differentiation (termed neogenesis) thus confirming the significance of RLCs as a pathophysiologically relevant renal progenitor cell niche [12]. In previous experiments we found that renin-positive RLCs were recruited from their classical juxtaglomerular (JG) position in the afferent arterioles of the adult kidney to differentiate into intraglomerular mesangial cells during the proliferative stage after acute mesangiolysis [8]. In this model of selective, antibody mediated mesangial cell depletion RLCs descendants differentiated exclusively into intraglomerular mesangial cells. However, since the mesangial injury model is induced by an anti-mesangial serum it could be assumed that limited damage develops in glomerular ECs or podocytes. Thus, the possibility remains that upon EC injury, RLCs may regenerate intraglomerular ECs. This is a feasible option for several reasons. During nephrogenesis RLCs develop not only into renin-producing JG cells but also in glomerular, vascular, tubular and interstitial cells representing altogether almost 10% of the adult kidney [12, 13]. The plasticity of RLCs persists in adulthood and is featured by reversible recruitment of renin-producing cells upstream in the afferent arterioles after chronic stimulation of renin production [14, 15]. In addition, the replacement of mesangial cells by RLCs upon glomerular damage replicates a developmental process since mesangial cells partially originate from RLCs [12, 13]. In turn, RLCs are largely descendants of stromal FoxD1-expressing cells in the developing kidney [16, 17]. Interestingly, it has been claimed that renal ECs may also arise from FoxD1 precursor cells during embryogenesis [18]. Lastly, we found that the renin-producing RLCs express angiogenic factors necessary for the integrity of the renal capillary endothelium [19].

Therefore, we hypothesized that RLCs may contribute not only to mesangial but also to EC repopulation after glomerular injury. To prove this hypothesis, we subjected triple-transgenic mice with pulse-labelled RLCs in the afferent arterioles to a severe yet reversible EC injury using our concanavalin-A/anti-concanavalin-A model.

## Materials and methods

### Animals

Triple-transgenic inducible mRen-rtTAm2/LC1/LacZ reporter mice were used to fate-map RLCs. In these animals the renin-producing cells and their descendants are labelled by the LacZ gene product β-galactosidase (β-gal) after doxycycline administration [8]. All procedures were prospectively approved by the local authorities (Approval No DD24.1-5131/394/34 by Landesdirektion Sachsen) and performed in accordance to the *DIRECTIVE 2010/63/EU* of the European Parliament. The experimental animals did not display any apparent adverse clinical signs during the course of the studies. Score sheet for humane endpoints was defined before starting experiments; however threshold for application was not reached for any animal.

### Doxycycline and enalapril administration

mRen-rtTAm2/LC1/LacZ mice received doxycycline (2 mg/ml) plus enalapril (10mg/kg) via drinking water for 16 days, starting at 8–12 weeks of age to induce permanent labelling of RLCs in the afferent arterioles. Enalapril was added to increase the renin transcription and thus the efficacy of the labelling as already described [8]. A washout period of at least 7 days was allowed before the onset of experimental EC injury.

### Experimental EC injury (EC model)

EC injury was induced using anti-concanavalin-A model [5, 9, 10]. Briefly, *Canavalia ensiformis* lectin concanavalin A was administered in the left renal artery allowing it to attach to the endothelial surface layer. Subsequent application of rabbit anti-concanavalin-A antiserum (SeqLab, Germany) causes immune-complex formation and complement mediated EC injury in the left kidney. Diseased mice were euthanized either on day 1 (n = 5) or day 7 (n = 10). A control group (n = 5, baseline day 0) received the same volume of saline and euthanized afterwards. For euthanasia, isoflurane (Baxter, Germany) anaesthetized animals were perfused with 4% paraformaldehyde (PFA) solution. Left kidneys were split in half, fixed in 4% PFA solution overnight at 4°C and embedded in paraffin or in NEG-50 frozen section medium (Thermo Fisher Scientific, Germany).

### Visualization of RLCs

To detect RLCs enzymatic β-gal staining or β-gal immunostaining was performed. For the enzymatic approach, 6-μm cryotome-sections were fixed in acetone and incubated in staining solution (staining buffer containing MgCl_2_, 5mM K_4_Fe(CN)_6_, 5mM K_3_Fe(CN)_6_ and 5-bromo-4-chloro-3-indolyl-β-D-galactopyranoside solved in dimethylformamide) for 6 hours at 37°C [8, 20].

### Immunostaining

Immunostainings and Acid fuchsin orange G (AFOG) staining were performed on paraffin-embedded 4-μm kidney sections as previously described [5, 8, 19]. Antibodies were diluted in 1% BSA/TBS. Primary antibodies were incubated overnight at 4°C, secondary antibodies and Avidin D-conjugated horseradish peroxidase were incubated for 2 hours at room temperature. All immunofluorescence samples were counterstained with the nuclear marker 4′,6-diamidino-2-phenylindole (DAPI) and mounted with mowiol mounting medium (Sigma Aldrich, Germany). Endogenous peroxidase activity was suppressed with 3% hydrogen peroxide solution. Samples were counterstained with Hematoxylin (Vector Laboratories, CA), dehydrated and mounted with Entellan (Merck Millipore, Germany). The following antibodies were used: WT-1 (Abcam, ab202639), nephrin (Progen, gp-n2), PDGFRβ (Abcam, 91066), α8-integrin (R&D, baf4076), CD31 (Dianova, dia310), ERG (Abcam, ab196149), β-galactosidase (Abcam, 9361), renin (R&D, af4277), CD45 (abcam 64100).

### Microscopy and histological assessment

Confocal images were acquired using an inverted Leica SP5 CLSM equipped with AOBS and HCX PL APO lambda blue 63.0x1.40 oil immersion objective. Additionally, Olympus Spinning Disc equipped with Olympus Plan Apo 60x /1.42 oil immersion objective was used. Fluorescence signal was sampled by sequential excitation/detection to minimize signal crosstalk. Histological assessment was performed using FIJI assisted densitometry. For analysis of glomerular morphology the whole renal cortex was screened to capture superficial, midcortical and juxtamedullary glomeruli.

### 3D reconstruction

β-gal/α8-integrin immunostaining was performed on serial 6-μm cryotome sections. After Axio Scan.Z1 (Zeiss, Germany) assisted acquisition, a stack of equal sized aligned images was reconstructed using FIJI “TrakEM2” plugin. Structure of individual glomeruli was rendered using image visualization and analysis software Imaris 8.3.1 (Bitplane, Switzerland).

### Statistical analysis

Statistical analysis was performed using one-way ANOVA with Bonferroni post-hoc testing (Graph Pad 5, Graph Pad Software Inc, CA). Results are presented as mean ± standard error (SEM). P<0.05 was considered significant.

## Results

### Reversible EC injury (EC model)

**Fig 1:**
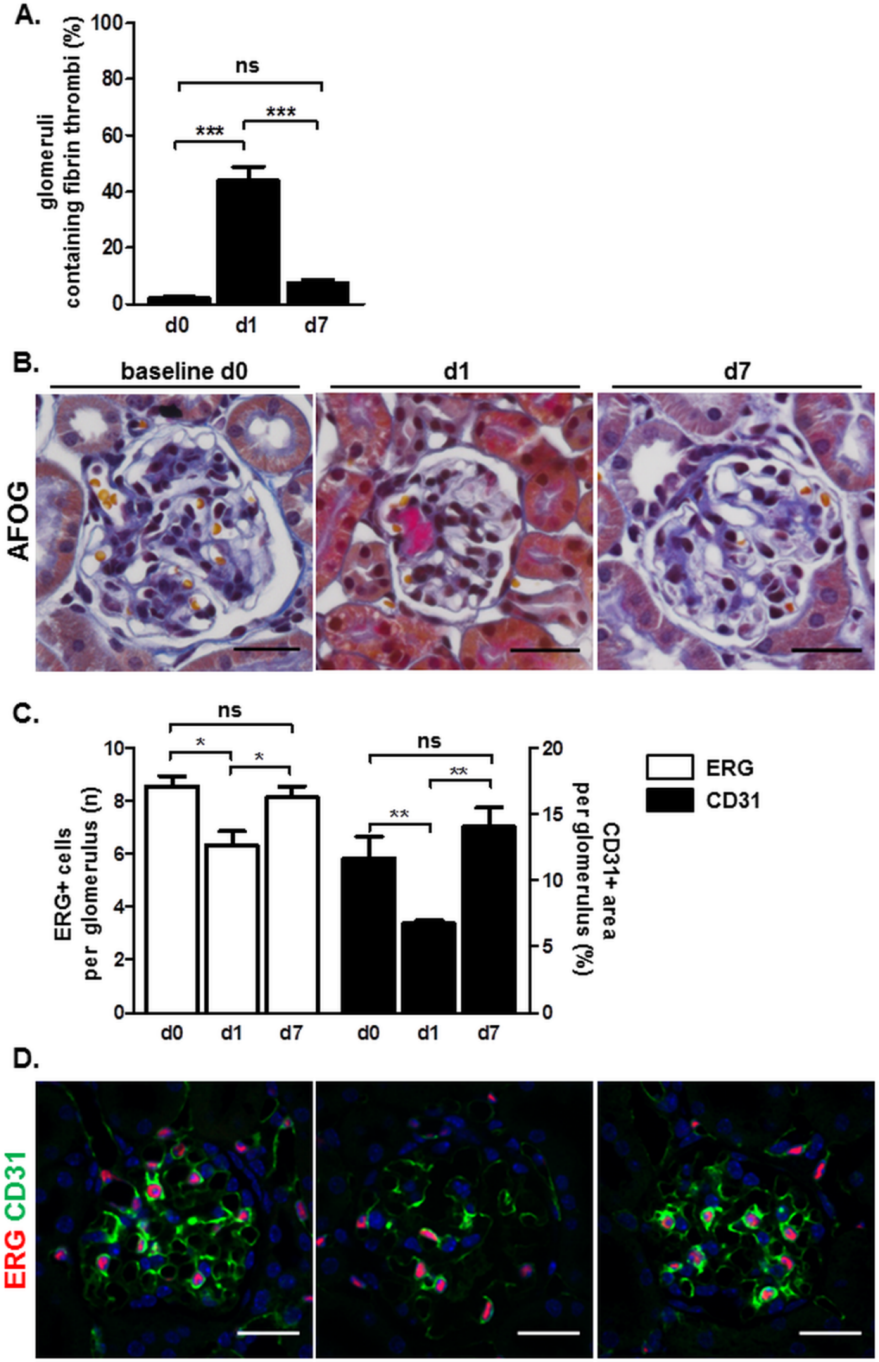
Reversible glomerular EC damage in mRen-rtTAm2/LC1/LacZ mice during thrombotic microangiopathy-like injury model. Mice were analyzed at baseline (day 0), day 1, and day 7 following disease induction. A. Quantification of intraglomerular fibrin thrombi (pinkish) as revealed by Acid Fuchsin Orange G (AFOG) staining of kidney slices. Data are presented as mean ± SEM, n = 5/5/10 for day 0 /day 1 /day 7, respectively. ***p<0.001, ns- not significant by one-way ANOVA; B. Representative AFOG staining images in the course of the EC model. Scale bars correspond to 25 μm; C. Quantification of glomerular EC depletion and injury on kidney slices by erythroblast transformation-specific related gene (ERG) and platelet endothelial cell adhesion molecule-1 (CD31) staining, respectively. Data are presented as mean ± SEM, n = 5/5/10 for day 0 /day 1 /day 7, respectively. *p<0.05, **p<0.01, ns- not significant by one-way ANOVA; D. Representative ERG/CD31 co-staining images in the course of the EC model. 4′,6-diamidino-2-phenylindole (DAPI) was used as a nuclear marker. Scale bars correspond to 25 μm.

Acute thrombotic microangiopathy-like microvascular damage was induced in the left kidneys of triple-transgenic mRen-rtTAm2/LC1/LacZ mice via concanavalin/anti-concanavalin administration, as previously reported [5, 9, 10]. In this reversible model, glomerular endothelial injury peaks 1 day after left renal artery injection of concanavalin/anti-concanavalin followed by a regenerative phase up to at least day 7. The model is considered to cause transient EC dysfunction and EC depletion. AFOG staining revealed glomerular fibrin thrombi on day 1 after disease induction (Fig 1A and 1B). Glomerular EC number, assessed by erythroblast transformation-specific related gene (ERG) immunostaining, was quantified at baseline (day 0), 1 day, and 7 days following model induction (Fig 1C and 1D). In healthy mice average EC number was 8.6 per glomerular cross-section. We observed a marked reduction to 6.3 glomerular ECs in diseased animals on day 1. ECs were fully restored to 8.4 cells per glomerular cross-section 7 days after disease induction. The EC surface marker platelet endothelial cell adhesion molecule-1 (CD31) confirmed the marked reduction of glomerular endothelial cells on day 1 following disease initiation and subsequent restoration to baseline levels on day 7, as revealed by immunofluorescence staining (Fig 1C and 1D). These findings of glomerular EC injury with subsequent regeneration are consistent in terms of magnitude and time course with our previous reports [5, 9, 10].

### RLCs invade the glomerulus during endothelial repair

**Fig 2:**
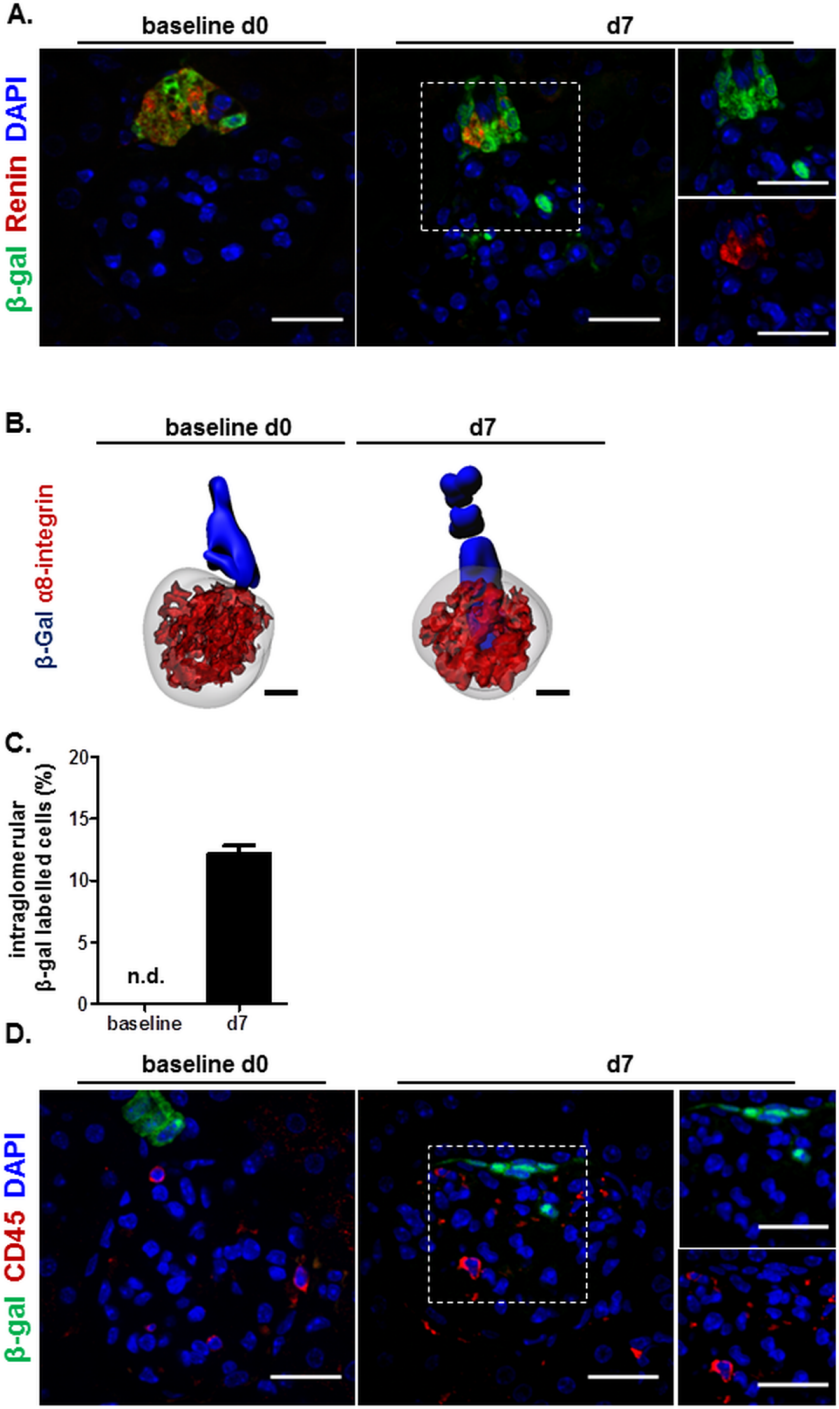
RLCs invade the glomerulus during EC model. A. Intraglomerular RLCs tagged by β-gal, but negative for renin appear in the regenerative phase of the EC model (day 7). Representative confocal microscopy images for day 0 and day 7 of β-gal/renin co-stained kidney slices. 4′,6-diamidino-2-phenylindole (DAPI) was used as a nuclear marker. The channels for green (β-gal) and red (renin) fluorescent signals in the dashed square on day 7 are separately shown in the small right panels. Scale bars correspond to 25 μm; B. Representative 3D reconstruction of glomeruli and β-gal labelled RLCs (blue) on day 0 and day 7 of the EC model. The mesangial cell marker α8-integrin (red) was used to visualize the glomeruli. Scale bars correspond to 20 μm; C. Quantification of glomeruli with tufts containing β-gal expressing RLCs in the regenerative phase of the EC model (day 7). Data are presented as mean ± SEM, n = 5/10 for day 0 (baseline) /day 7, respectively. n.d.—not detectable; D. The intraglomerular RLCs observed during the EC model are not of hematopoietic origin. Representative confocal microscopy images for day 0 and day 7 of β-gal/CD45 (hematopoietic marker) co-stained kidney slices. 4′,6-diamidino-2-phenylindole (DAPI) was used as a nuclear marker. The channels for green (β-gal) and red (CD45) fluorescent signals in the dashed square on day 7 are separately shown in the small right panels. Scale bars correspond to 25 μm.

We next investigated whether during EC model RLCs migrate into the glomerulus and contribute to endothelial replenishment. For this purpose, permanent LacZ (β-gal) labelling of the renin-producing RLCs in the afferent arterioles was induced in adult healthy mRen-rtTAm2/LC1/LacZ mice [8]. β-gal positivity was observed exclusively in the afferent arterioles before EC model induction (Fig 2A). However, in the regenerative phase of the EC model (day 7) a subset of β-gal positive cells was detected within the glomerular tufts on kidney sections. These results could be confirmed by three-dimensional reconstructions of serial kidney slices (Fig 2B). Quantitative evaluation revealed that 12.9% of all glomeruli showed immigration of RLCs on day 7 (Fig 2C). The intraglomerular RLCs did not express renin in contrast to RLCs at their classical position in the afferent arterioles (Fig 2A, middle panel). The intraglomerular RLCs were also negative for CD45 demonstrating that they are not of hematopoietic origin (Fig 2D). Thus, in agreement with earlier studies of our group utilizing a mesangial cell injury model [8] we found that RLCs are capable of repopulating injured glomeruli also during the EC model.

### RLCs selectively differentiate to intraglomerular mesangial cells during EC model

**Fig 3:**
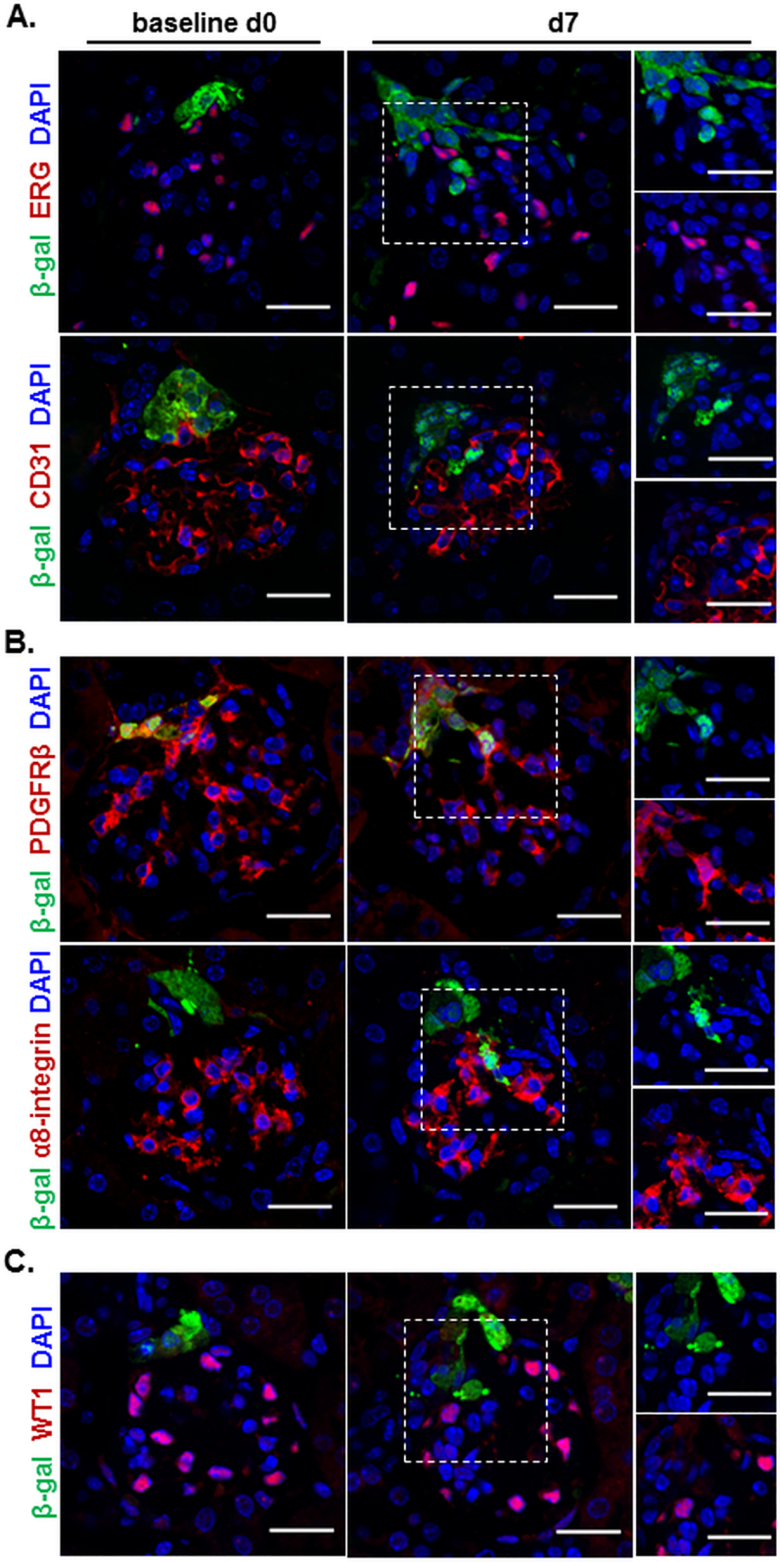
RLCs selectively differentiate to intraglomerular mesangial cells during EC model. A. Representative confocal microscopy images for day 0 and day 7 of β-gal and the EC markers ERG (upper panels) or CD31 (lower panels) co-stained kidney slices; B. Representative confocal microscopy images for day 0 and day 7 of β-gal and the mesangial cell markers PDGFβR (upper panels) or α8-integrin (lower panels) co-stained kidney slices; C. Representative confocal microscopy images for day 0 and day 7 of β-gal/WT1 (podocyte marker) co-stained kidney slices. 4′,6-diamidino-2-phenylindole (DAPI) was used as a nuclear marker throughout. The channels for green (β-gal) and red (corresponding cell marker) fluorescent signals in the dashed squares on day 7 are separately shown in the small right panels. Scale bars correspond to 25 μm.

Since loss of glomerular endothelium is a hallmark of our EC model we hypothesized that RLCs replace the damaged glomerular ECs. However, no intraglomerular β-gal labelled RLCs were found positive for EC markers (CD31 or ERG) on day 7 corresponding to the regenerative phase of the model (Fig 3A). Subsequently we were interested to identify the phenotype of the repopulating intraglomerular RLCs. Since it was already known that RLCs may give rise to mesangial cells upon glomerular injury [7, 8] we first examined this possibility in our EC model. Indeed, intraglomerular RLCs stained positive for the mesangial cell markers platelet derived growth factor receptor β (PDGFRβ) and α8-integrin on day 7 after EC model induction (Fig 3B). Three-dimensional reconstructions confirmed that all intraglomerular RLCs were α8-integrin-positive (Fig 2B, right). In addition, we also co-stained for podocytes to find no expression of podocyte markers in intraglomerular RLCs on day 7 of the EC model (Fig 3C). These results clearly showed that even after severe EC injury the repopulating RLCs develop selectively to intraglomerular mesangial cells.

### Mesangial cells display considerable damage in the course of the EC model

**Fig 4:**
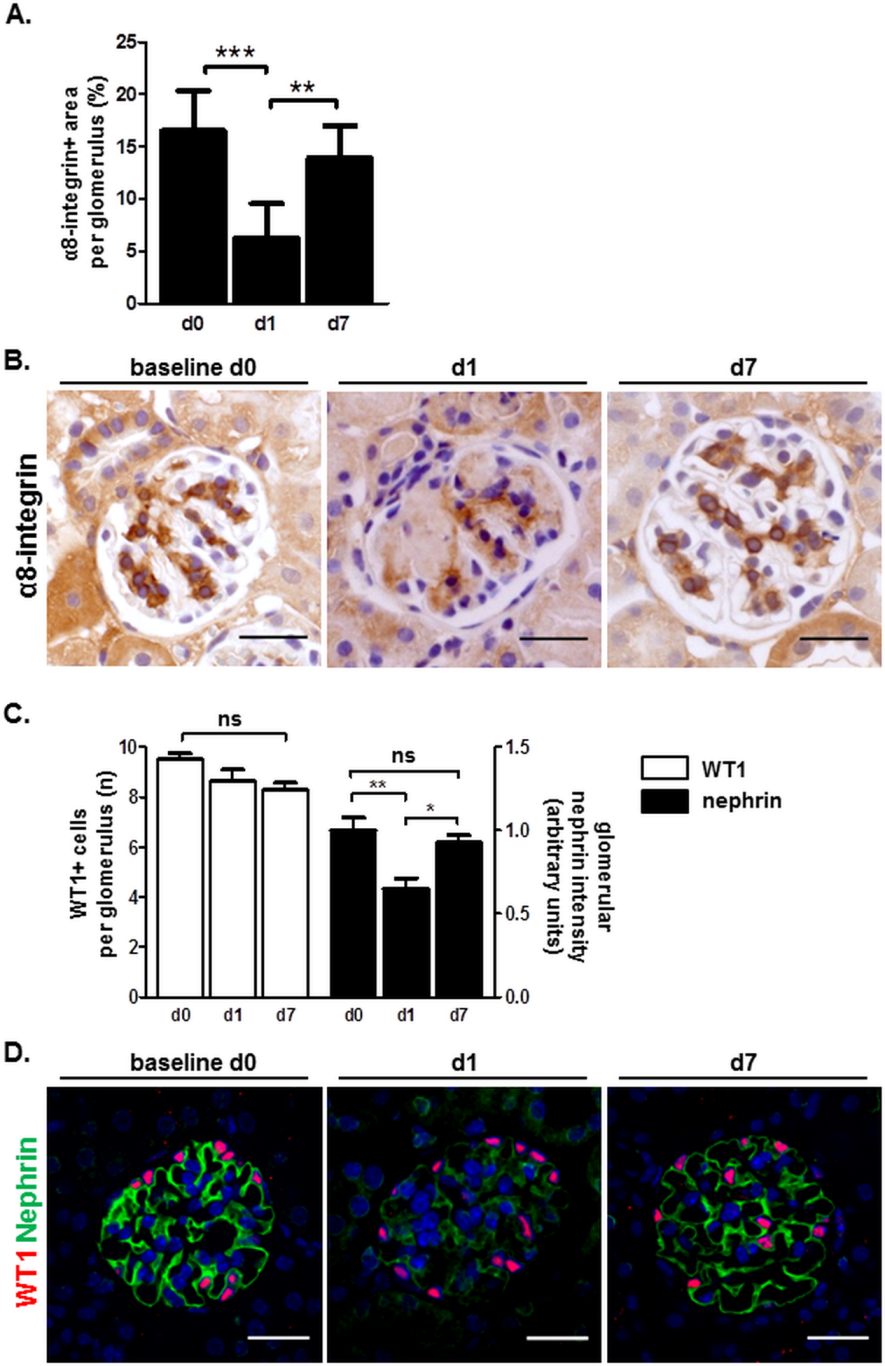
Mesangial cells and not podocytes display considerable transient damage in the course of the EC model. Mice were analyzed at baseline (day 0), day 1, and day 7 following disease induction. A. Quantification of glomerular mesangial injury by α8-integrin (mesangial cell marker) staining of kidney slices. Data are presented as mean ± SEM, n = 5/5/10 for day 0 /day 1 /day 7, respectively. **p<0.01, ***p<0.001 by one-way ANOVA; B. Representative α8-integrin staining images in the course of the EC model. Scale bars correspond to 25 μm; C. Quantification of podocyte depletion and injury on kidney slices by WT1 and nephrin staining, respectively. Data are presented as mean ± SEM, n = 5/5/10 for day 0 /day 1 /day 7, respectively. *p<0.05, **p<0.01, ns- not significant by one-way ANOVA; D. Representative WT1/nephrin co-staining images in the course of the EC model. 4′,6-diamidino-2-phenylindole (DAPI) was used as a nuclear marker. Scale bars correspond to 25 μm.

To provide mechanistic support for our findings we addressed the possibility that glomerular mesangial cells are damaged during our EC model. Mesangial injury as assessed by decreased glomerular α8-integrin staining developed within 1 day after the start of the EC model (Fig 4A and 4B). Glomerular α8-integrin immunostaining restored to baseline levels on day 7. Interestingly no significant podocyte depletion was observed (as assessed by WT1 nuclear staining) in the course of the EC model although reversible podocyte injury became discernable by decreased nephrin staining on day 1 (Fig 4C and 4D). Altogether these findings indicated that mesangial cell injury and mild podocyte impairment transiently occurred during the EC model. The mesangial damage appeared to serve as a stimulus for the extraglomerular RLCs in the afferent arterioles to invade the glomerulus and acquire mesangial phenotype therein.

## Discussion

This study was performed to address the possibility that renin-producing RLCs in the afferent arterioles of the adult kidney may act as progenitors for glomerular ECs after renal endothelial injury. We used a triple-transgenic mouse strain for fate tracing. The same mice had already been successfully used to demonstrate that renin precursor cells from the afferent arterioles migrate into the glomeruli to replace damaged mesangial cells in the proliferative phase after acute mesangiolysis [8]. We had also described the EC model as a clinically relevant approach to study kidney-specific endothelial injury, in which endothelial repair via endogenous renal cells occurs within a week [5, 9, 10]. Both depletion and damage of glomerular ECs were observed in the course of our disease model. Consistent with our data received with the same mouse strain after mesangial injury, we could observe that RLCs repopulate the damaged glomeruli after severe EC injury. Despite complete restoration of capillary tuft structure and glomerular EC number, repopulating RLCs did not express EC markers, indicating that they do not to contribute to endothelial replenishment. This finding was somewhat unexpected because our previous experience clearly suggested that regeneration utilizes developmental differentiation programs [8, 12] and that the RLCs preserve their differentiating capacity in the adult kidney [14, 15]. During nephrogenesis both RLCs and renal ECs seem to have a common precursor cell pool namely the FoxD1 cells [18]. There is strong evidence that RLCs arise from the FoxD1 precursor cell pool [16, 17]. On the other hand the development of renal endothelium from FoxD1 progenitors is still under discussion because there are reports arguing against the FoxD1 origin of renal ECs [21]. While a contradiction about the embryonic progenitor cell pool of renal ECs remains, we could definitely conclude that in our severe EC injury model RLCs do not contribute to glomerular endothelial regeneration. Nevertheless, we found that RLCs from the afferent arteriole migrate into the glomerular tuft in the regenerative phase after EC injury. These intraglomerular RLCs could be identified exclusively as mesangial cells. This observation is in line with earlier findings by us and other groups that in mouse models of mesangiolysis/mesangioproliferative glomerulonephritis and FSGS RLCs migrate from JG position into the glomeruli and differentiate into mesangial cells [6–8]. Thereby they lose renin expression and acquire mesangial phenotype (undergo transdifferentiation). Thus, the RLCs in the afferent arterioles appear to be a pivotal progenitor cell niche that is induced upon different modes of glomerular damage to give rise to mesangial cells. Such interpretation is particularly feasible because it corroborates the importance of an embryonic process namely the differentiation of RLCs into mesangial cells as an underlying mechanistic principle in the regeneration of the adult kidney after injury [12, 13].

The observed mesangial injury in our EC model would provide a trigger for the RLCs in the afferent arterioles to replace the injured intraglomerular mesangial cells. Since we used an antibody-mediated endothelial-specific mechanism to induce renal injury, the mesangial cells are likely to be damaged secondary to the endothelial disease. Lining the glomerular capillary loops ECs lie in close contact to the mesangial cells. The interaction between endothelial and mesangial cells is decisive for the structural and functional integrity of the glomerulus. For instance, PDGFB produced by EC is a master regulator of mesangial cell development. Endothelial-specific PDGFB deficiency during nephrogenesis leads to formation of single looped malfunctioning glomeruli due to failure to attract mesangial cell progenitors [22]. PDGFRβ is expressed on mesangial cells and its deletion leads to a similar phenotype thus validating the close endothelial-mesangial crosstalk [23]. Moreover EC stress, such as shear stress or hypoxia, leads to diminished PDGFB production [24, 25]. Therefore, it is plausible to assume that in our EC model the primary endothelial damage led to impaired endothelial-mesangial cell communication and caused indirect mesangial injury. The extent of mesangial injury in the EC model was about half of that in our mesangial cell injury model [8] and corresponded to the different degree of intraglomerular recruitment of RLCs during the regenerative phase in both models.

Using a similar mouse line and a model of FSGS Lichtnekert *et al*. found that RLCs may replace podocytes in diseased glomeruli [6]. However, we did not observe podocyte replenishment by RLCs when applying renal injury models [8, 12]. A plausible explanation for this discrepancy is that podocytes are not affected in the models we used. Consistent with such interpretation we found only transient moderate podocyte injury, but no podocyte depletion during the EC model used herein.

## Conclusions

In summary, we reported here that renin-positive RLCs from the afferent arterioles repopulate damaged glomeruli in a thrombotic microangiopathy-like kidney-specific EC injury model. Despite injury and depletion of glomerular ECs during the model, RLCs did not participate in the regeneration of the glomerular endothelium but rather replaced damaged glomerular mesangial cells. The mesangial cells appear to be indirectly damaged during the course of the EC model, thus providing a mechanistic explanation for the intraglomerular RLC recruitment. These findings further support the emerging concept that in the adult kidney RLCs function as a specific precursor pool for mesangial cells which can be mobilized upon mesangial cell injury.
